# Error in Methods

**DOI:** 10.1001/jamanetworkopen.2025.52604

**Published:** 2025-12-11

In the Original Investigation titled “Evaluation of Patients’ Adverse Events During Contact Isolation for Vancomycin-Resistant *Enterococci* Using a Matched Cohort Study With Propensity Score,”^1^ published March 10, 2022, there was an error in the Methods. The threshold for statistical significance should have been “*P* < .05.” This article has been corrected.^1^
